# Transcriptomic Response of *Clonostachys rosea* Mycoparasitizing *Rhizoctonia solani*

**DOI:** 10.3390/jof9080818

**Published:** 2023-08-02

**Authors:** Zhan-Bin Sun, Shu-Fan Yu, Man-Hong Sun, Shi-Dong Li, Ya-Feng Hu, Han-Jian Song

**Affiliations:** 1 School of Light Industry, Beijing Technology and Business University, Beijing 100048, China; 2 Institute of Plant Protection, Chinese Academy of Agricultural Sciences, Beijing 100193, China

**Keywords:** biocontrol, comparative transcriptomic analysis, differentially expressed genes, fungal plant pathogens, mycoparasitism

## Abstract

*Clonostachys rosea* is an important mycoparasitism biocontrol agent that exhibits excellent control efficacy against numerous fungal plant pathogens. Transcriptomic sequencing may be used to preliminarily screen mycoparasitism-related genes of *C. rosea* against fungal pathogens. The present study sequenced and analyzed the transcriptome of *C. rosea* mycoparasitizing a Basidiomycota (phylum) fungal pathogen, *Rhizoctonia solani*, under three touch stages: the pre-touch stage, touch stage and after-touch stage. The results showed that a number of genes were differentially expressed during *C. rosea* mycoparasitization of *R. solani*. At the pre-touch stage, 154 and 315 genes were up- and down-regulated, respectively. At the touch stage, the numbers of up- and down-regulated differentially expressed genes (DEGs) were 163 and 188, respectively. The after-touch stage obtained the highest number of DEGs, with 412 and 326 DEGs being up- and down-regulated, respectively. Among these DEGs, ABC transporter-, glucanase- and chitinase-encoding genes were selected as potential mycoparasitic genes according to a phylogenetic analysis. A comparative transcriptomic analysis between *C. rosea* mycoparasitizing *R. solani* and *Sclerotinia sclerotiorum* showed that several DEGs, including the tartrate transporter, SDR family oxidoreductase, metallophosphoesterase, gluconate 5-dehydrogenase and pyruvate carboxylase, were uniquely expressed in *C. rosea* mycoparasitizing *R. solani*. These results significantly expand our knowledge of mycoparasitism-related genes in *C. rosea* and elucidate the mycoparasitism mechanism of *C. rosea*.

## 1. Introduction

*Rhizoctonia solani* (teleomorph: *Thanetophorus cucumeris*) is a destructive necrotrophic fungal pathogen that belongs to Basidiomycota, Agaricomycetes, Ceratobasidiales, Ceratobasidiaceae [1]. The hyphal color of *R. solani* changes from whitish to brown as the hyphae develop from young to mature [2]. The hyphae then form into sclerotia, which are the main infection source of *Rhizoctonia solani*, and exhibit strong resistance to a hostile environment [3,4,5]. *Rhizoctonia solani* infects plants belonging to 32 families, such as Malvaceae, Fabaceae, Poaceae, Brassicaceae and Amaranthaceae. *Rhizoctonia solani* infection causes severe plant diseases and losses [6,7,8].

Chemical control is a widely used method to control plant diseases caused by *R. solani*. However, the following reasons limit the use of pesticides to some extent: environmental pollution and easily generated pesticide resistance to *R. solani*. Biocontrol is another effective control method that has attracted much attention due to its advantages of being green, safe and sustainable. Microorganisms such as *Trichoderma* sp., *Clonostachys rosea*. and *Lecanicillium muscarium* are important biocontrol agents [9,10,11]. Biocontrol mycoparasites generally target living fungi that parasitize fungal pathogens as a type of biocontrol agent. Several mycoparasites containing *Trichoderma* strains (*T. asperellum*, *T. koningii*, *T. virens*, *T. harzianum*, and *T. atroviride*) and *Clonostachys rosea* have exhibited desirable control effects against plant diseases caused by *R. solani* [10,11,12,13,14,15]. In addition to screening more biocontrol mycoparasites, examining the mycoparasitism molecular mechanism is another an important pathway to further improving control efficacy against plant diseases caused by *R. solani*.

The screening and identification of mycoparasitism-related genes are the basis for investigating the molecular mechanisms of mycoparasitism. Many methods may be used to acquire mycoparasitism-related genes, such as subtractive hybridization, transcriptomic analysis, cDNA microarray, etc. Among these methods, transcriptomic analysis is a crucial and common method. Investigating alterations in the expression levels of genes from mycoparasites during the process of mycoparasitic *R. solani* using transcriptomic analysis has helped further examine mycoparasitism-related genes. The screening of mycoparasitism-related genes using transcriptomic analysis has primarily focused on *Trichoderma* species. Numerous mycoparasitism-related genes have been examined from *T. virens* and *T. atroviride* using transcriptomic analysis when these *Trichoderma* species mycoparasitized *R. solani* [16,17,18].

*Clonostachys rosea* is an excellent biocontrol mycoparasite that protects against numerous fungal plant pathogens [19]. Numerous mycoparasitism-related genes have been screened from *C. rosea* against *Sclerotinia sclerotiorum*, *Fusarium graminearum*, *Botrytis cinerea* and *Helminthosporium solani* according to transcriptomic sequencing and analysis [20,21,22,23]. However, there are no reports of the screening of mycoparasitism-related genes from *C. rosea* against *R. solani*. Because taxonomic differences exist between *R. solani* and the other four pathogens in the phylum, the types of mycoparasitism-related gene may distinguish *C. rosea* against *R. solani* against the other reported four pathogens. Therefore, it is important to examine mycoparasitism-related genes from *C. rosea* against *R. solani*.

*Clonostachys rosea* 67-1 is an effective strain that exhibits desirable inhibition of *R. solani* and control efficacy against rice sheath blight disease caused by *R. solani* [24]. To screen mycoparasitism-related genes from *C. rosea* against *R. solani*, the present study collected the mycelium of *C. rosea* against *R. solani* at three stages: pre-touch, touch and after-touch. We investigated differentially expressed genes from *C. rosea* during the process of mycoparasitizing *R. solani*. We compared the transcriptomes of *C. rosea* against other fungal pathogens and analyzed specific mycoparasitism-related genes in *C. rosea* against *R. solani*. The results of the present study significantly expand our knowledge of mycoparasitism-related genes in *C. rosea* and elucidate the mycoparasitism mechanism of *C. rosea*.

## 2. Materials and Methods

### 2.1. Strains

*Clonostachys rosea* 67-1 was originally isolated from a vegetable yard in Hainan Province, China. *Clonostachys rosea* 67-1 and the pathogen *R. solani* were preserved and cultivated on PDA (potato dextrose agar) plates.

### 2.2. Sample Collection

*Clonostachys rosea* 67-1 and *R. solani* were cultivated on PDA plates at 26 °C for 10 d. Sterilized cellophane was placed on the surface of the PDA plates, and equally sized mycelium plugs of *C. rosea* 67-1 and *R. solani* were taken and placed at opposite positions on the PDA plates and cultivated at 26 °C. Three stages were selected for sampling to reflect the mycoparasitism process of *C. rosea*. The pre-touch stage (PT) occurred when the distance between *C. rosea* 67-1 and *R. solani* was 0.5 cm. The touch stage (T) occurred when *C. rosea* 67-1 and *R. solani* grew together. The after-touch stage (AT) occurred when *C. rosea* 67-1 and *R. solani* had crossed growth of 0.5 cm (Figure S1). The mycelium of *C. rosea* 67-1 from each treatment was collected and used for RNA extraction. *Clonostachys rosea* 67-1 growth alone at each stage was used as a control. All of the treatments were performed with three replications.

### 2.3. cDNA Library Construction and Transcriptome Sequencing

The total RNA of the mycelium in each sample was extracted. An Agilent 2100 bioanalyzer was used to detect the quality of the extracted RNAs. Oligo (dT) magnetic beads were used to enrich the mRNAs of each sample. The enriched mRNAs were randomly broken by divalent cations in NEB fragmentation buffer.

The mRNAs were reverse transcribed into the first-strand cDNA using an M-MuLV reverse transcriptase system, and then, the second-stand cDNA was synthesized using a DNA polymerase I system. The purified cDNA was subjected to end repair, the addition of adenine to the 3′ end and adapter connection to generate 250-300 bp cDNA fragments using AMPure XP beads (Beckman Coulter, High Wycombe, UK). cDNA libraries were constructed via the amplification of suitable cDNA fragments. After quality control, the cDNA libraries were sequenced using the Illumina NovaSeq 6000 platform (Illumina Inc., San Diego, CA, USA) at Novogene Corporation Inc.

### 2.4. Transcriptome Data Processing

The raw sequencing data contained few reads that had adapters or low sequencing quality. To ensure the reliability of transcriptome data analysis, the raw sequencing data were filtered to acquire clean data as follows. Reads that contained adaptor sequences and that contained N (unknown bases) and low-quality reads were removed. All reads were submitted to the NCBI sequence read archive (SRA) database to obtain accession numbers. The clean sequences were mapped onto the genome of *C. rosea* 67-1 to obtain their location in the genome using HISAT2 software v2.0.5 [25].

### 2.5. DEG Analysis

The expression levels of the genes were calculated using FPKM (expected number of fragments per kilobase of transcript sequence per million base pairs sequenced), which corrected the sequencing depth and gene length [26,27]. DESeq2 software (1.20.0) was used to screen differentially expressed genes (DEGs) in *C. rosea* mycoparasitizing *R. solani* at three different stages [28]. Genes with significant differential expression were confirmed as having an expression fold change of greater or less than 2 and a p value of less than 0.05. Cluster analysis was performed between all treatments to reflect the relationships of samples or genes. Gene Ontology (GO) and Kyoto Encyclopedia of Genes and Genomes (KEGG) pathway enrichment analyses of DEGs were performed using clusterProfiler software (3.8.1) based on the hypergeometric distribution principle.

To screen for potential mycoparasitism-related genes, three types of DEG encoding ABC transporters, glucanases and serine proteases from *C. rosea* were used to perform phylogenetic analysis with corresponding genes with known function from *Trichoderma* strains. The selected corresponding genes of each type are effective against *R. solani* (Table 1). Sequences of each type of DEG were aligned using CLUSTAL_X, and phylogenetic analysis was performed using MEGA 6.0 with the maximum likelihood method under the Kimura two-parameter model [29,30].

### 2.6. Comparative Transcriptome Analysis

Because the taxonomic status of *R. solani* is quite different from that of other commonly known fungal pathogens, investigating the unique DEGs from *C. rosea* mycoparasitizing *R. solani* compared to other fungal pathogens helps further our understanding of the molecular mechanism of *C. rosea* mycoparasitism. We previously performed transcriptomic analysis of *C. rosea* mycoparasitizing *S. sclerotiorum*. Therefore, *S. sclerotiorum* was used as a typical Ascomycota pathogen for comparative transcriptome analysis in the present study. The unique DEGs and their pathways involved in *C. rosea* mycoparasitizing *R. solani* compared to mycoparasitizing *S. sclerotiorum* were analyzed.

### 2.7. Quantitative Real-Time PCR Validation

Twelve DEGs were randomly selected and their expression levels were detected through quantitative real-time PCR for validation. The primer pairs of the 12 DEGs were designed using Primer Premier 6.0 software (Table S1).

Quantitative real-time PCR was performed using a CFX96 Real-Time PCR Detection System, using programs of 95 °C for 30 s; 40 cycles of 95 °C for 5 s; and 60 °C for 30 s. The expression levels of the 12 DEGs were calculated using the 2^−ΔΔCt^ method. Elongation factor and actin were used as internal reference genes. Each experiment was performed with three replications.

## 3. Results

### 3.1. Transcriptome Sequencing and Data Processing

**Figure 1:**
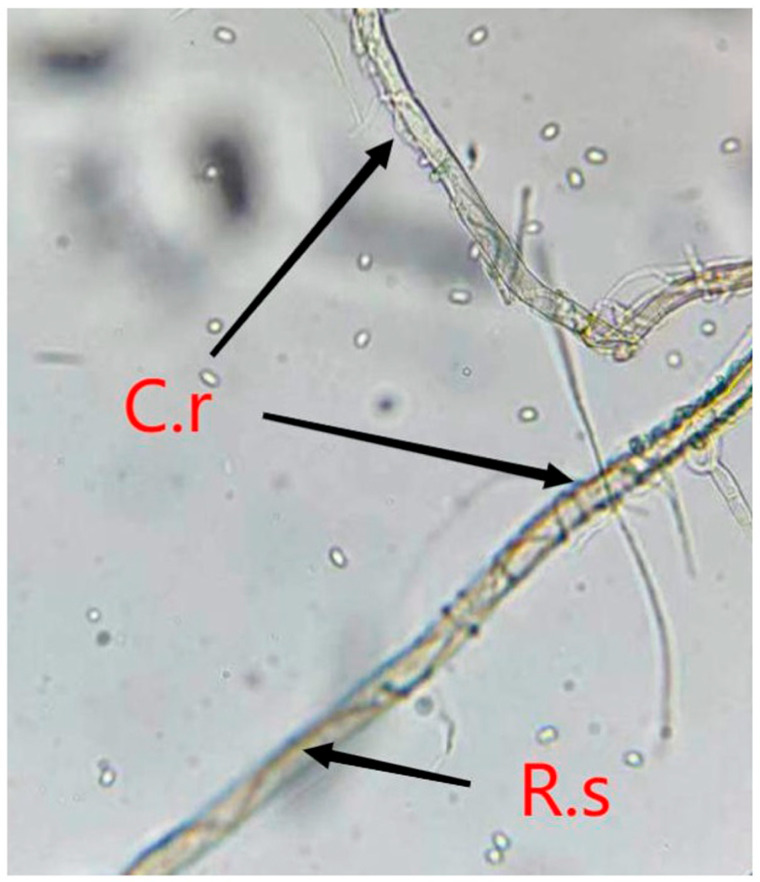
Mycoparasitism of *Clonostachys rosea* on *Rhizoctonia solani* under microscope observation. C.r. means *Clonostachys rosea*; R.s. represents *Rhizoctonia solani*.

The microscope observation showed that the mycelium of *C. rosea* was tightly intertwined with *R. solani* at both the T and AT stages, which indicates that *C. rosea* was mycoparasitizing *R. solani* at the time of touch (Figure 1).

After transcriptome sequencing, the Q20 and Q30 of each sample were higher than 96.71% and 91.43%, respectively. The average clean read after data filtration was 45,863,230. The mapping rate to the genome of *C. rosea* between treatments ranged from 95.01 to 97.33, with an average rate of 96.22%. The sequencing data were submitted to SRA, under the following accession numbers: SRR22822402, SRR23032134, SRR22987521, SRR22981867, SRR22980386, SRR22963970, SRR22953580, SRR22947653, SRR22942454, SRR22937423, SRR22894263, SRR22891490, SRR22891348, SRR22890531, SRR22888634, SRR22876798, SRR22875421 and SRR22859027.

### 3.2. DEG Analysis

Hundreds of genes were differentially expressed during the process of *C. rosea* mycoparasitizing *R. solani*. Most of these DEGs belonged to hypothetical proteins. At the pre-touch stage, 154 and 315 genes were up- and down-regulated, respectively, during *C. rosea* mycoparasitism. The highest expression levels of up-regulated DEGs were annotated to polyketide synthase, MFS transporter and acetylxylan esterase-like protein. The down-regulated DEGs with the highest expression fold changes were annotated to aldehyde dehydrogenase and SGNH hydrolase-type esterase.

The numbers of downregulated DEGs during the touch stage of *C. rosea* mycoparasitism were lower than those in the pre-touch stage, with 163 and 188 genes being up- and down-regulated, respectively. Up-regulated DEGs such as D-isomer-specific 2-hydroxyacid dehydrogenase, S-layer homology domain-containing protein, chaperonin 10-like protein and NADPH-p450 reductase had the highest fold changes in expression. Among the downregulated DEGs, arabinogalactan endo-1,4-beta-galactosidase, GCN5-N-acetyltransferase and glycosyl hydrolase family 61-domain-containing protein exhibited higher fold changes in expression.

Among the three mycoparasitism stages, the numbers of DEGs in the after-touch stage were higher than those in the other stages. A total of 412 and 326 DEGs were up- and down-regulated, respectively. Genes annotated to aldehyde dehydrogenase, NAD-dependent epimerase/dehydratase and alpha/beta hydrolase protein exhibited the highest expression fold changes of the up-regulated DEGs. For the down-regulated DEGs, the highest expression fold changes of genes were annotated to cytochrome P450, GMC oxidoreductase and L-amino acid oxidase.

### 3.3. GO Enrichment Analysis

**Figure 2:**
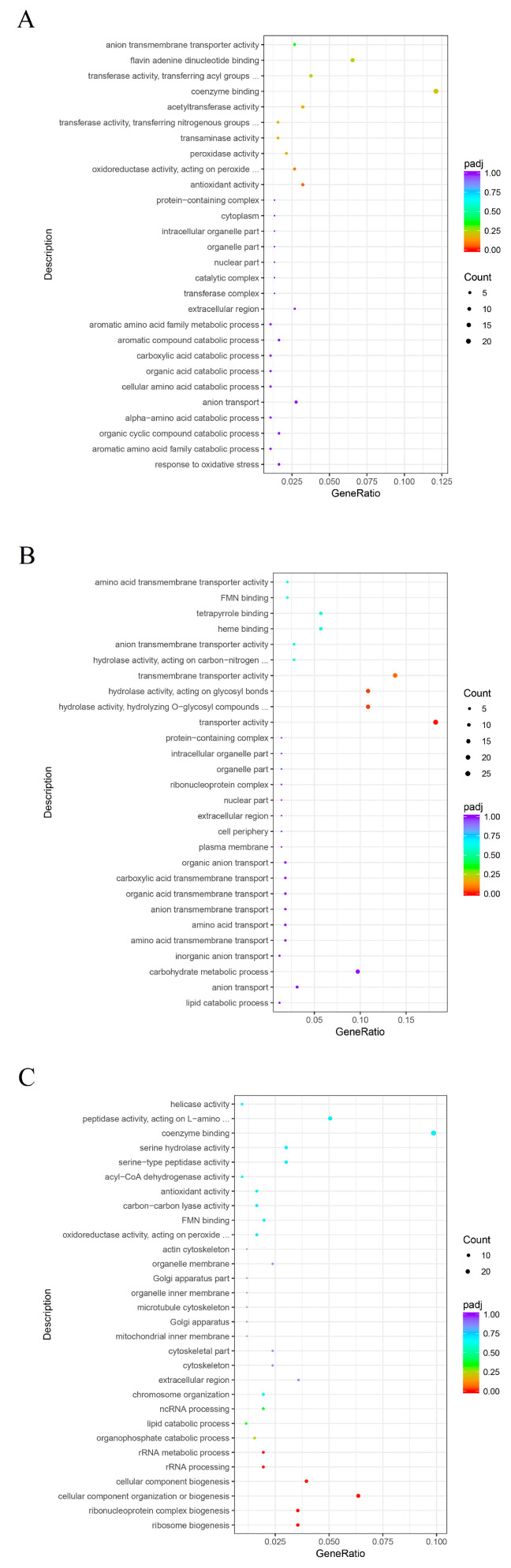
Gene ontology (GO) categories of the differentially expressed genes. (**A**) Pre-touch stage; (**B**) touch stage; (**C**) after-touch stage.

DEGs from all three mycoparasitic stages were assigned to three categories: biological process, cellular components and molecular function. Most DEGs in the pre-touch stage were annotated to carbohydrate metabolic processes and the regulation of gene expression in the biological process category. DEGs annotated to coenzyme binding and transporter activity were the dominant groups in the molecular function category. Most DEGs in the cellular component category were annotated to the extracellular region (Figure 2A).

The dominant groups in the biological process and molecular function categories in the touch stage were similar to those in the pre-touch stage, carbohydrate metabolic processes and transporter activity. The numbers of DEGs in each group of cellular component categories were equal (Figure 2B). The dominant groups among the three categories in the after-touch stage were quite different from those in the other two mycoparasitic stages. The dominant group in the biological process category was the regulation of gene expression, the regulation of macromolecule metabolic processes and small molecule metabolic processes. Organelle part and intracellular organelle part were the main groups in the cellular components category. The dominant groups in the molecular function category were coenzyme binding, ATP binding and adenyl ribonucleotide binding (Figure 2C).

### 3.4. KEGG Enrichment Analysis

**Figure 3:**
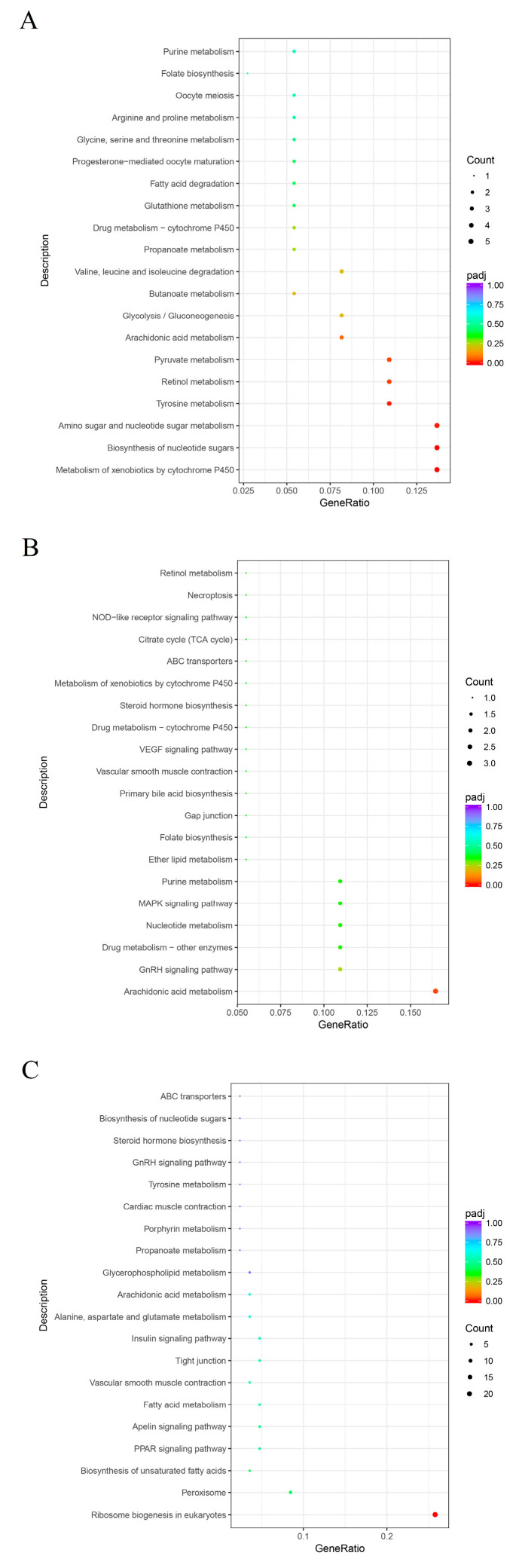
KEGG analysis of the differentially expressed genes. (**A**) Pre-touch stage; (**B**) touch stage; (**C**) after-touch stage.

The dominant KEGG pathways among the three mycoparasitic stages were different. The main pathways in the pre-touch stage were the metabolism of xenobiotics by cytochrome P450, the biosynthesis of nucleotide sugars, and amino sugar and nucleotide sugar metabolism (Figure 3A). The dominant pathways in the touch stage were arachidonic acid metabolism and drug metabolism—other enzymes (Figure 3B). The pathways of ribosome biogenesis in eukaryotes and peroxisomes were dominant in the after-touch stage (Figure 3C).

### 3.5. Screening of Potential Mycoparasitism-Related Genes

**Figure 4:**
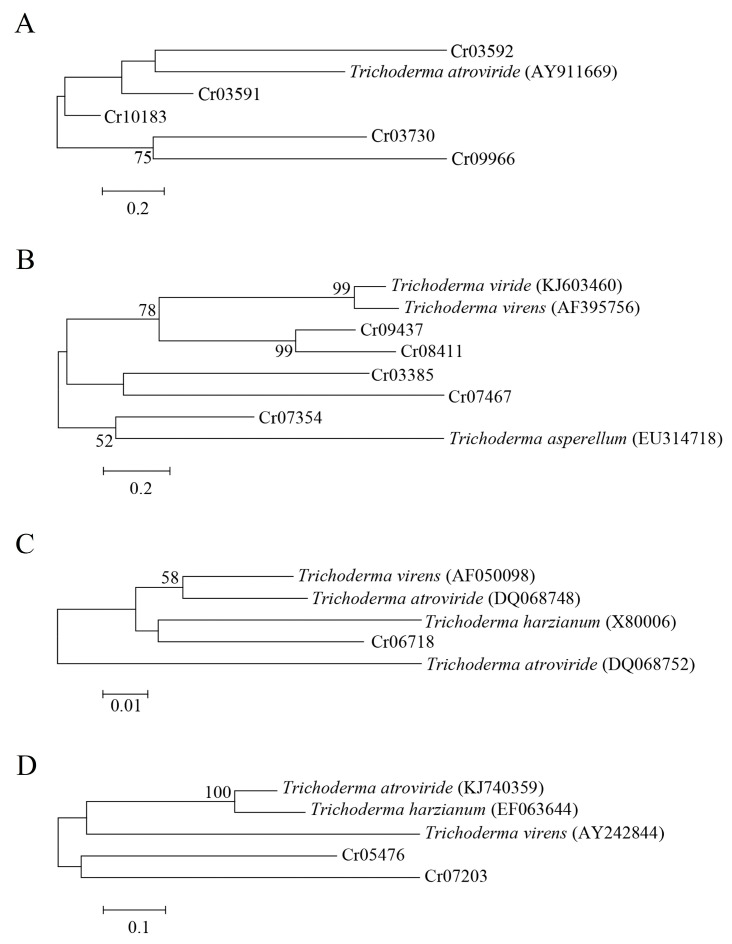
Phylogenetic analysis of genes from *Clonostachys rosea* using the maximum likelihood method. Numbers at the nodes indicate the bootstrap values of 1000 bootstraps. Bars represent sequence divergence. (**A**) ABC transporter; (**B**) glucanases; (**C**) chitinase; (**D**) serine protease.

Five ATP-binding cassette (ABC) transporters, five glucanases, two serine proteases and one chitinase were differentially expressed in *C. rosea* mycoparasitizing *R. solani*. Phylogenetic analysis showed that the ABC transporter-encoding gene *Taabc2* was closely related to Cr03592, which may be a mycoparasitism-related gene compared to other ABC transporter-encoding genes (Figure 4A). For glucanases and chitinase, phylogenetic analysis showed that Cr07354 and Cr06718 were most closely related to exo-β-1,3-glucanase and chitinase, respectively, and may be used as potential mycoparasitism-related genes for further functional verification (Figure 4B,C). The serine protease-encoding genes Cr05476 and Cr07203 from *C. rosea* were far from all of the serine protease genes from the *Trichoderma* species according to the phylogenetic analysis (Figure 4D). Therefore, the function of the two serine protease genes in mycoparasitism needs further investigation.

### 3.6. Comparative Transcriptomic Analysis

Compared to *C. rosea* mycoparasitizing *S. sclerotiorum* [20], several DEGs were unique during the process of *C. rosea* mycoparasitizing *R. solani* at three stages. At the pre-touch stage, genes encoding tartrate transporter protein, synaptic vesicle transporter and phosphoacetylglucosamine mutase were uniquely expressed. Among these unique DEGs, the gene encoding phosphoacetylglucosamine mutase was involved in the biosynthesis pathways of nucleotide sugars, amino sugar and nucleotide sugar metabolism.

The DEGs encoding formylmethionine deformylase, pyruvate carboxylase and victoriocin were unique at the after-touch stage. The pyruvate carboxylase gene participated in multiple pathways, including the citrate cycle, pyruvate metabolism, the biosynthesis of amino acids and carbon metabolism. Several DEGs, including gluconate 5-dehydrogenase, SDR family oxidoreductase and metallophosphoesterase, were unique at the touch stage. Genes encoding SDR family oxidoreductases were involved in the peroxisome pathway.

### 3.7. Quantitative Real-Time PCR Validation

**Figure 5:**
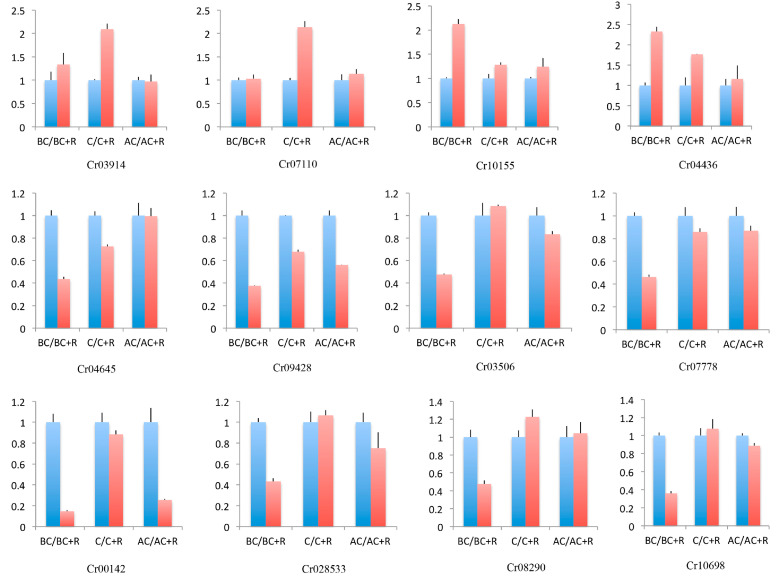
Quantitative real-time PCR to validate the expression levels of DEGs. The *X*-axis represents different sampling stages, and the *Y*-axis represents relative expression levels of DEGs. The blue column represents *Clonostachys rosea* only, and the red column represents added *Rhizoctonia solani*. Error bars indicate the standard deviation (SD) of three replications.

The expression levels of the 12 selected DEGs were consistent with the transcriptomic analysis, which indicates that the expression levels of the DEGs from the transcriptome were reliable (Figure 5).

## 4. Discussion

The screening of DEGs is the basis for exploring the molecular mechanism of mycoparasitism. Transcriptome sequencing and analysis are commonly used to screen DEGs. Although several fungal plant pathogens, such as *S. sclerotiorum*, *F. graminearum*, *B. cinerea* and *H. solani*, have been used as target pathogens during *C. rosea* mycoparasitism to screen for DEGs using transcriptome sequencing, all of these pathogens belong to the phylum Ascomycota. Therefore, screening DEGs from *C. rosea* mycoparasitizing other taxonomic pathogens is helpful for investigating the molecular mechanism of mycoparasitism to provide new insights. The present study selected *R. solani* because its taxonomic status is quite different from that of the other reported pathogens. Three stages, pre-touch, touch and after-touch, were used to represent the early, middle and final stages of *C. rosea* mycoparasitism, respectively. DEGs were screened in three mycoparasitic stages using transcriptomic sequencing and analyzed. The unique DEGs were also analyzed in *C. rosea* mycoparasitizing *R. solani* compared to *S. sclerotiorum*.

Transcriptomic sequencing and analysis revealed that numerous genes were differentially expressed during *C. rosea* mycoparasitization of *R. solani*. Among these DEGs, several genes contained ABC transporters, heat shock protein 70, transcription factors, polyketide synthase, cell wall-degrading enzymes such as chitinase, β-glucanase and serine proteases, which were previously reported to have mycoparasitic ability against fungal plant pathogens using gene function analysis [31,32,33,34,35,36]. The present study used ABC transporters, β-glucanase, serine proteases and chitinase as potential mycoparasitic-related genes to perform phylogenetic analysis for the following reasons: there are differences between *R. solani* and other Ascomycetes fungal plant pathogens, and only a few genes had verified functions as biocontrol agents against *R. solani*. The above four types of gene were previously reported to be related to *R. solani* in *Trichoderma* species [37,38,39,40]. Several genes of each type were differentially expressed during *C. rosea* mycoparasitization of *R. solani* in the present study. Therefore, we analyzed which DEGs were closely related to the function-known mycoparasitism genes using phylogenetic analysis, and the potential mycoparasitism genes in *C. rosea* against *R. solani* were also screened.

Cell wall-degrading enzymes commonly have biocontrol ability against fungal plant pathogens because these enzymes can degrade the cell walls of fungal pathogens. There are three different types of cell wall-degrading enzyme: chitinase, glucanase and protease. *Clonostachys rosea* has been reported to be able to produce the above three types of cell wall-degrading enzyme [41]. Pasqualetti found that *C. rosea* IG119 could produce chitinolytic enzymes [42,43]. The activities of chitinase produced by *C. rosea* 67-1 were enhanced after overexpression of the chitinase-encoding gene [34]. Moreover, the activities of β-1,3-glucanase produced by *C. rosea* were induced under the presence of fungal pathogen cell walls [44]. Serine protease was purified from *C. rosea* and exhibited the ability to degrade the nematode cuticle [45]. The deletion or overexpression of genes encoding cell wall-degrading enzymes reduces or increases the biocontrol ability of biocontrol agents. Five glucanase genes, two serine protease genes and one chitinase gene were differentially expressed in *C. rosea* 67-1 mycoparasitizing *R. solani*. Genes encoding chitinases, glucanases and serine proteases are involved in *Trichoderma* against *R. solani*. Co-expression of the β-1,3- and β-1,6-glucanase genes in *T. virens* improved the bioprotection of cotton seedlings against *R. solani* [38]. The expression of the β-1,3-glucanase gene from *Trichoderma* spp. in *indica* and *japonica* rice cultivars reduced the disease severity of *R. solani* [46]. The disruption and overexpression of chitinase-encoding gene *cht42* could significantly decrease and enhance the biocontrol ability of *T. virens* against cotton seedling diseases caused by *R. solani* compared with the wild strain [47]. Phylogenetic analysis revealed that one β-glucanase gene, Cr07354, and one chitinase gene, Cr06718, were most closely related to the reported genes, respectively, which indicates that Cr07354 and Cr06718 in *C. rosea* 67-1 have potential against *R. solani*. However, both serine protease genes were far from the reported genes in the phylogenetic analysis, and their function in mycoparasitism needs further exploration.

ABC transporters function in detoxication [48]. The deletion and complementation of the ABC transporter-encoding gene *abcG5* in *C. rosea* IK726 influenced xenobiotic tolerance, which may be due to *abcG5*’s involvement in detoxifying the *Fusarium* mycotoxin zearalenone and other fungicides [49]. ABC transporters also play crucial roles in biocontrol. Deletion of the ABC transporter-encoding gene *Taabc2* in *T. atroviride* strongly reduced the ability to overgrow, inhibit and degrade the mycelia of *R. solani*. The mutant significantly reduced the protective effect of tomato plants infected with *R. solani* [37]. Five ABC transporter genes were used to perform phylogenetic analysis, and only one ABC transporter gene, Cr03592, was most closely related to the reported gene, which suggests that Cr03592 was a mycoparasitism-related gene against *R. solani*.

Additionally, genes associated with up-stream signal transduction, such as as G protein; transcribing, such as transcription factor; and down-stream mycoparasitism-related metabolite synthesis, such as polyketide synthase, were all differentially expressed during *C. rosea* mycoparasitizing *R. solani*. As an important signal transduction protein, G protein could regulate the transduction of mycoparasitism-related signals through signaling pathways, and then, influence the control ability of biocontrol agents. G protein was involved in fungal growth, development and secondary metabolism, as well as virulence to the host. Several studies have reported that G protein plays important roles in biocontrol among different biocontrol agents. The disruption of the G protein α subunit-encoding gene *thga3* in *T. harzianum* could dramatically affect its mycoparasitic ability against *R. solani* [50]. In *T. atroviride*, the deletion of the G protein α subunit-encoding gene *tga1* could lose its mycoparasitic ability against *R. solani*, *B. cinerea*, and *S. sclerotiorum*, as well as reducing chitinase activity in the mutant. Moreover, overexpressed *tga1* in *T. atroviride* could improve the mycoparasitic ability against *R. solani* [51,52]. The disruption of another G protein α subunit-encoding gene, *tga3*, in *T. atroviride* could influence its mycoparasitic ability against *R. solani* and *B. cinerea*, infection structures and chitinase activity [53]. In other *Trichoderma* species, G protein α subunit had similar effect on mycoparasitic ability. The deletion of the G protein α subunit-encoding gene *tgaA* in *T. virens* could influence the mycoparasitic ability to *S. rolfsii* [54]. Besides *Trichoderma*, the deletion of the G protein α subunit-encoding gene *MrGPA1* in *Metarhizium robertsii* could reduce the virulence in *Galleria mellonella* larvae [55].

Transcription factors are trans-acting proteins that can be activated by signaling pathways, thereby regulating the expression levels of target genes. Transcription factors are highly conserved in fungus and associated with numerous biological processes such as fungal growth, development and tolerance to environmental stress. Transcription factors have been reported to be involved in biocontrol in both mycoparasitism and entomopathogenic fungal agents. In biocontrol mycoparasites, deletion of transcription factor-encoding genes in *C. rosea* could reduce the virulence and mycoparasitic ability to nematode and *S. sclerotiorum*, respectively [33,56]. In *T. virens*, the disruption of the transcription factor-encoding gene *pacC* could influence the mycoparasitic capacity against *R. solani* and *S. rolfsii* [57]. Similarly, the roles of transcription factors in biocontrol have been found in *Coniothyrium minitans*, and disruption of the transcription factor-encoding gene could affect the mycoparasitic ability against *S. sclerotiorum*, as well as the activities of chitinase and β-1,3-glucanase [58]. In entomopathogenic fungal agents, the absence of the transcription factor-encoding genes *MaPacC* and *Mrap1* could influence the virulence of *M. acridum* and *M. rileyi* in *Locusta migratoria manilensis* and *Spodoptera litura* larvae [59,60]. The disruption of the transcription factor-encoding genes *Bbmcm1* and *BbHapX* in *Beauveria bassiana* could affect the virulence against *G. mellonella* larvae [61,62].

Polyketide synthases were able to synthesize different types of polyketide, among which antibiotics and toxins had the potential to act against fungal plant pathogens. Polyketide synthase-encoding genes have been reported to be involved in biocontrol. The deletion of the polyketide synthase-encoding gene *pks29* in *C. rosea* could influence the antagonistic ability against *B. cinerea* and biocontrol capacity against fusarium foot rot in barley. The absence of another polyketide synthase-encoding gene, *pks22*, in *C. rosea* could influence the production of Clonorosein A and B, which exhibit antifungal activity against *F. graminearum* and *B. cinerea* [35]. The deletion of the polyketide synthase-encoding gene *pks4* in *T. reesei* could influence the antagonistic abilities against *Rhizoctonia solani*, *Sclerotinia sclerotiorum*, and *Alternaria alternate* [63]. Similarly, the disruption of the polyketide synthase-encoding gene could affect the virulence of *B. bassiana* against beet armyworms [64].

Comparative transcriptomic analysis revealed that several DEGs were unique at three stages during *C. rosea* mycoparasitizing *R. solani* compared to *S. sclerotiorum*. Although studies of these DEGs that participate in the biocontrol process are lacking, these genes were differentially and uniquely expressed during *C. rosea* mycoparasitization of *R. solani*. Therefore, exploring the function of these unique DEGs in *C. rosea* mycoparasitism is important for extending the mycoparasitism-related gene pool of *C. rosea*. Future work should focus on investigating the function of these DEGs during *C. rosea* mycoparasitization of *R. solani* using gene knockout, complementation or overexpression.

## 5. Conclusions

Transcriptomic sequencing and analysis were performed on *C. rosea* mycoparasitizing *R. solani* under three touch stages. A total of 154 and 315 genes, 163 and 188 genes, and 412 and 326 genes were up- and down-regulated at the pre-touch stage, touch stage, and after-touch stage, respectively. ABC transporter-, glucanase- and chitinase-encoding genes were screened as potential mycoparasitism genes during *C. rosea* against *R. solani* using phylogenetic analysis. DEGs such as SDR family oxidoreductase, metallophosphoesterase and tartrate transporter were unique in *C. rosea* mycoparasitizing *R. solani* compared to that mycoparasitizing *S. sclerotiorum* according to our comparative transcriptomic analysis. These results expand the types of mycoparasitism-related gene in *C. rosea* and elucidate the mycoparasitism mechanism of *C. rosea*.

## Figures and Tables

**Table 1:** Genes used for phylogenetic analysis.

Gene Annotation	Gene Name	Isolate	Accession No.
ABC transporter	*Taabc2*	*T. atroviride*	AY911669
β-1,3-glucanase	*TvBng2*	*T. virens*	AF395756
Exo-β-1,3-glucanase	*tag83*	*T. asperellum*	EU314718
β-1,3-glucanase	-	*Trichoderma* spp.	KJ603460
Subtilisin-like serine protease	*TghSS42*	*T. ghanense*	KJ740359
Subtilisin-like protease	*SS10*	*T. harzianum*	EF063644
Serine protease	*tvsp1*	*T. virens*	AY242844
Chitinase	*chit42*	*T. virens*	AF050098
Chitinase	*chit33*	*T. harzianum*	X80006
Chitinase	*chi18-10*	*T. atroviride*	DQ068748
Chitinase	*chi18-13*	*T. atroviride*	DQ068752

## Data Availability

Not applicable.

## Supplementary Materials

The following supporting information can be downloaded at: https://www.mdpi.com/article/10.3390/jof9080818/s1, Figure S1: Photos of *Clonostachys rosea* mycoparasitizing *Rhizoctonia solani* on plates; Table S1: Primers used for quantitative real-time PCR.
