# Supplementary material for: Transcriptomic Response of Clonostachys rosea Mycoparasitizing Rhizoctonia solani
Source: J Fungi (Basel). 2023 Aug 2;9(8):818. doi: 10.3390/jof9080818 (PMC10455738; doi:10.3390/jof9080818)
Supplement: Supplementary file 1 [file jof-09-00818-s001.zip › jof-2537695-supplementary.pdf]

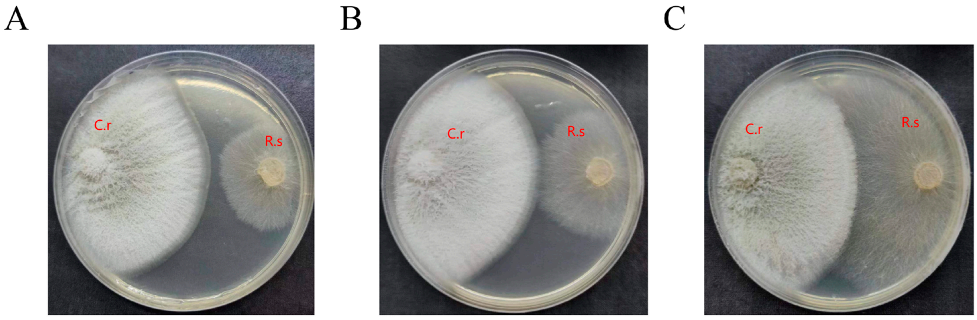

Figure S1. Photos of *Clonostachys rosea* mycoparasitizing *Rhizoctonia solani* on plates. (**A**) pre-touch stage; (**B**) touch stage; (**C**) after-touch stage. C.r means *Clonostachys rosea*; R.s represents *Rhizoctonia solani*.

.

Table S1. Primers used for quantitative real-time PCR

Primers	Sequence (5'-3')
EF1F	TCGATGTCGCTCCTGACT
EF1R	AGCGTGACCGTTTATTTGA
ACTF	CAAGTCCAACCGTGAGAA
ACTR	GTAAATAGGGACAACGTGAGT
Cr07110F	CCGTGCAGGACAGGATAA
Cr07110R	CCAATGGAGTTGATGAGGG
Cr10155F	GGAACGGTCCAGGTCAACA
Cr10155R	AGGGAACTGCACTCGAAAGA
Cr04436F	TCTTCTCCTTCGCCTCCG
Cr04436R	CACCTACCATACCGCACTCG
Cr04645F	GCCGACGCCTTCATTACA
Cr04645R	CAGTGCTCGTGAGTCCATC
Cr09428F	ATGGGCAGTTTATTATTCCG
Cr09428R	TTGGCCTTGTTGTTGACG
Cr03506F	TAGCCTTGTACCAGATGCC
Cr03506R	CCAGTAAACCTTGTCTCCCT
Cr03914F	TCAACTFGAAATCGGACTGG
Cr03914R	TGAAGCGGACGTAGAAGG
Cr07778F	AATCCTGGCTCAGCTTGT
Cr07778R	CACTATTGCCTTCGTACTTTC
Cr00142F	GTGGTGGCAGCCAGAATG
Cr00142R	CGAGGGTCAAGTCGGAGAT
Cr028533F	AGTCCATCAAGCAAGGTGTT
Cr028533R	TGAGCGGACTCGTCAACC
Cr08290F	CCGTGGAGGACCCGATTGA
Cr08290R	TGGCTTTCCGGCATTTCT
Cr10698F	TTACCAAGAATGGGAAGC
Cr10698R	AAGACGAGTGGCGATGAC
